# Assessing the Impact of Anthropogenically Modified Land Uses on Wetland Health: Case of Witbank Dam Catchment in South Africa

**DOI:** 10.3390/w16162287

**Published:** 2024-08-13

**Authors:** Sylvester Mpandeli, Stanley Liphadzi, Chengetanai Mabhaudhi, Tafadzwanashe Mabhaudhi, Luxon Nhamo

**Affiliations:** 1 Water Research Commission of South Africa, Lynwood Manor, Pretoria 0081, South Africa; 2 Department of Environmental, Water and Earth Sciences, Tshwane University of Technology (TUT), Pretoria 0029, South Africa; 3 Faculty of Science, Engineering and Agriculture, University of Venda, Thohoyandou 0950, South Africa; 4 Centre for Transformative Agricultural and Food Systems (CTAFS), School of Agricultural, Earth and Environmental Sciences, University of KwaZulu-Natal, Pietermaritzburg 3209, South Afric; 5 Centre on Climate Change and Planetary Health, London School of Hygiene and Tropical Medicine (LSHTM), London WC1E 7HT, UK; 6 United Nations University Institute for Water, Environment and Health, Richmond Hill, ON L4B 3P4, Canada

**Keywords:** water quality, filtration, land use, wetland functions, nonpoint source pollution

## Abstract

Wetlands are critical ecological infrastructures that improve water quality, serve as habitat for fish and other aquatic life, accumulate floodwaters, and maintain surface water flow during dry periods. However, the health of wetlands has been compromised by anthropogenic activities that affect the constant supply of ecosystem services. This study assessed the impact of anthropogenically modified land use on wetland health in the Witbank Dam Catchment in South Africa, whose land use has been severely modified for agriculture and mining purposes. The study developed a model linking surface runoff generated in the catchment with land use and wetland typology to comprehend diffuse pollution from pollution-source land uses. Runoff data and related wetland spatial information were processed and analysed in a Geographic Information System (GIS) to estimate pollutants (agricultural nutrients and acid mine drainage) from runoff detained and released by wetlands. The analysis facilitated the assessment of the value of wetlands in enhancing water quality, as well as human and environmental health. The runoff volume from pollution-source land uses (urban areas, farmlands, and mining) was used to evaluate annual pollution levels. Wetland types are ranked according to their efficiency levels to filter pollutants. The assumption is that the difference between filtered and unfiltered runoff is the quantity of polluted runoff water discharged into the river system. The analysis has shown that 85% of polluted runoff generated in the catchment ends up in the river system. An important observation is that although wetlands have a substantial ability to absorb excess pollutants, they have finite boundaries. Once they reach their full holding capacity, they can no longer absorb any further pollutants. The excess is discharged into the river system, risking human and environmental health. This explains why the Limpopo River is heavily polluted resulting in the death of fish, crocodiles and other aquatic life.

## 1 Introduction

Wetlands, both natural and constructed, provide a variety of ecological and economic functions that include water quality improvement, flood protection, climate regulation, nutrient processing, carbon sequestration, groundwater recharge, shoreline stabilisation, habitat for aquatic life, aesthetics, and biological productivity, among other functions [1–3]. The importance and value of a wetland are determined by the worth of one or all of its functions to society and the environment [4]. Thus, wetlands provide worthy services to society and the environment that humankind may not appreciate, as 35% of the world’s wetlands were severely degraded between 1970 and 2015 [5,6]. Different types of wetlands provide distinct functions at different efficiency levels [7,8]. The recognition of the value of wetlands has witnessed an exponential increase in wetland research in recent years, particularly their effectiveness in providing nature-based solutions against the threats posed by climate change [7,9,10]. Research has developed various wetland-based water treatment smart technologies that are being used as green eco-technologies to treat water pollution [11–13]. Technological advances have made wetlands more valuable resource-recovery and crop production amenities [14,15].

The ability of wetlands to detain runoff and store water reduces the flow momentum and erosive potential of runoff, thereby reducing floods and land degradation [16]. This allows groundwater recharge, which in turn contributes to base flow and surface water availability during dry seasons [17]. During the period when a wetland detains runoff before its release, the water is purified by trapping sediments and retaining excess nutrients and other pollutants that are part of the runoff load [1,7]. During the storage period, the runoff load is dropped and settles on the wetland floor where these substances are often absorbed by plants and microorganisms in the soil or are transformed through denitrification and injected back into the atmosphere [18,19]. Wetlands are, therefore, rich in plant nutrients. By the time the water leaves the wetland, the filtration of nutrients and other chemical transformation processes would have removed much of the runoff pollutants, enhancing water quality and river health in the process [14]. Thus, healthy wetlands are biodiversity areas that enhance resilience to climate change and promote healthy environments [20]. This is supported by previous studies that have established that wetlands can store about 35% of terrestrial carbon dioxide, yet they cover only 9% of the planet’s surface [21,22]. Continued degradation of wetlands is, therefore, resulting in significant carbon dioxide and methane emissions that contribute to extreme weather events experienced today [23].

Therefore, understanding the interlinkages between human well-being and environmental health is at the centre of wetland management and their contribution to public health [24]. The human–wetlands–ecosystems nexus has attracted global attention in recent years due to the severity of wetland degradation which has undermined ecosystem services and exacerbated climate extremes like floods [25,26]. As a result, a wider recognition of the existing interlinkages between human well-being and environmental quality has been a topical subject of discussion in international environmental and climate change discourses [27,28]. This is based on the reality that wetlands are critical in enhancing human and environmental health and catalysing the realisation of Sustainable Development Goals (SDGs) [29,30], yet they have been extremely overexploited for food production and extraction of water resources to meet the requirements of a growing human population [31].

The sustainability of wetlands and their ability to continue providing essential services is diminishing at alarming rates [26,32]. These challenges have also seen a decline in public health and livelihoods, as access to water and sanitation remains a far cry for many [33]. Wetlands contribute immensely to the sustenance of life on earth, yet they have been perceived as the source of waterborne and other infectious diseases and a threat to sanitation [26,34,35]. This claim of unhealthy wetlands originates from poor upstream agricultural and mining activities that have degraded the wetlands [36]. This calls for the formulation of integrated and holistic environmental management strategies that simultaneously support the restoration and maintenance of the ecological attributes of wetlands and their benefits to people and the planet [26,37]. In the case of South Africa, over 65% of the country’s wetlands are classified as under threat and 48% of them are critically endangered [34]. This presents a critical outlook for a water-scarce country like South Africa. The country has enacted various governance structures to protect wetlands, but enforcement has been lacking, exposing wetlands to further degradation [37].

Considering the importance of wetlands and the rate at which they have degraded, several models and approaches have been developed to assess their economic and ecological value and the impact on human and environmental health as a result of their degradation [24,26,38]. Existing models tend to focus on nutrient and sediment retention [39–41], climate regulation and carbon sequestration [42,43], flood attenuation [44], groundwater recharge and shoreline stabilisation [45,46], among others. This study enhances the existing body of knowledge by developing a model that assesses wetland capacity, by type, to retain particulate and dissolved pollutants that form part of runoff load derived from anthropogenically modified landscapes. The model supports policy and decision-makers to understand the value of wetlands in regulating pollutants from cultivated lands and mines from being deposited into water courses. The model was piloted in the Witbank Dam Catchment (WDC), where water quality has significantly degraded due to massive land use changes that have been taking place in the catchment, including coal mining, extensive agriculture and rapid urbanisation [47]. The motivation is rooted in the understanding that the land use of a catchment determines the amount and quality of runoff water entering its water system [48,49]. The heavily polluted runoff load from the WDC has contributed to the Olifants River becoming one of the most polluted rivers in southern Africa [50], as several incidents of fish mortality have been recorded in the past years [51]. The study refers to runoff toxicants and nutrients to heavy metals and biocides from mines and urban areas and phosphates and nitrates from cultivated lands that form part of the runoff load [52]. These are also referred to as particulate and dissolved pollutants that form part of the runoff load [53].

Given the benefits of wetlands to people and the planet amidst overexploitation and degradation, this study developed a simplified model to guide the formulation of coherent strategies to protect and conserve the remaining healthy wetlands and revive the ones that have been degraded. This model was piloted in the WDC in the Upper Olifants Basin in South Africa. The catchment landscape is heavily modified for its rich and abundant agriculture and mineral resources [50]. The study determined the effectiveness of wetlands in removing pollutants from runoff water passing through anthropogenically modified land uses. Surface runoff is linked with land use area and wetland typology to estimate pollutant runoff generated in the catchment and discharged into the water system.

## 2 Materials and Methods

### 2.1 Description of the Study Area

**Figure 1:**
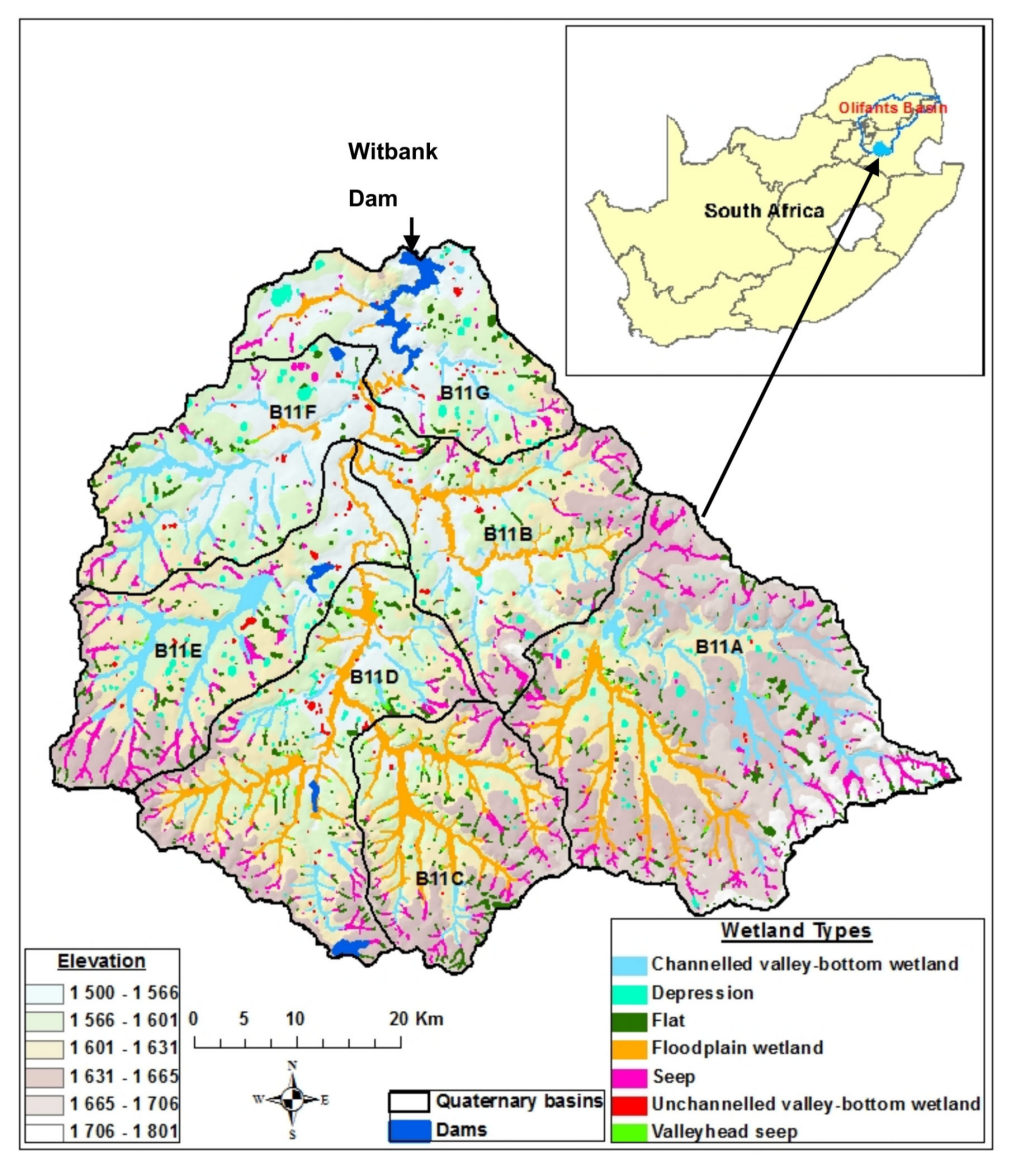
Location, elevation, and wetland types of the Witbank Dam Catchment. Source: Van Devente et al., 2020 [54].

The WDC covers an area of about 3500 km^2^ in the headwaters of the Olifants River, a major tributary of the Limpopo River Basin. The catchment has seven sub-basins (also known as quaternary basins (QB) in South Africa) as shown in Figure 1. The map (Figure 1) also shows the elevation of the WDC and its location in South Africa. The catchment is located upstream of the Witbank Dam in Mpumalanga Province. It has an average altitude of 1588 m above sea level, a mean annual precipitation of 689 mm, and a mean annual runoff of 125 × 10^6^ m^3^ a^*−*1^. There are over 2900 wetlands of different types in the catchment (Figure 1) [54]. There are abundant groundwater resources found in shallow weathered aquifers which serve as an important source of water in the catchment [55,56]. The geology consists of igneous and metamorphosed rocks. Granite is the dominant rock type, with common occurrences of dolerite intrusions, in the form of dykes and sills, and silicified sedimentary formations [57]. These rock formations favour the vast coal deposits in the catchment. Coal mining and associated industries are a major threat to water quality in Mpumalanga Province [57]. The Witbank coalfields are the largest conterminous coal mining area in South Africa [56].

The dominant soil types are moderately deep sandy to sandy clay loams [56]. The basin’s geology contributed to the formation of the catchment’s five major soil types, including cambic arenosols, chromic luvisols, chromic vertisols, orthic acrisols and rhodic ferrosols [58]. The land use in the catchment is predominantly agriculture (both irrigated and rainfed), improved and unimproved grassland, coal mining and mineral processing, bushland, urban and scattered rural settlements, and power generation (Figure 1). The catchment is densely populated, and the main source of livelihood is agriculture, which contributes to the high diffuse pollution. Therefore, the land area of the catchment has been heavily altered for agricultural and mining purposes [56,59]. About 38% of the catchment area is used for agriculture, placing its water resources at high risk of phosphate and nitrate contamination. The wetlands (Figure 1) are essential for water filtration and nutrient and sediment retention services at different efficiency capacities [59].

### 2.2 Land Use/Cover and Related Statistics

**Figure 2:**
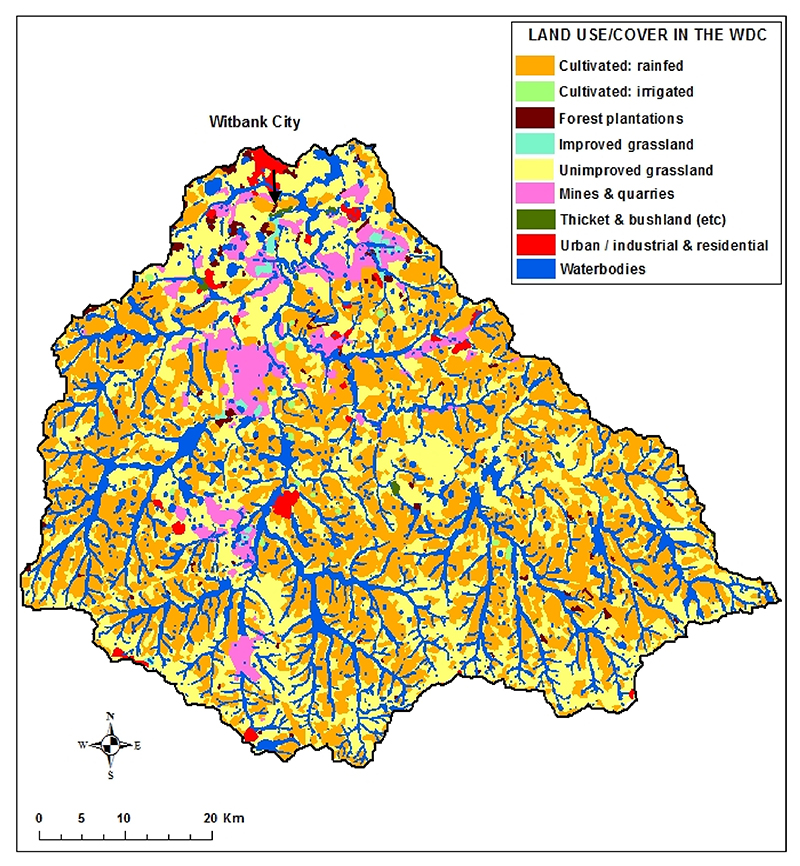
Land use/cover of the Witbank Dam Catchment.

The land use/cover map was extracted from the 2020 landcover map of South Africa obtained from the Department of Forestry, Fisheries, and Environment (DFFE), available at https://egis.environment.gov.za/sa_national_land_cover_datasets (accessed on 10 August 2024). We assumed that the more the natural land cover is altered for human activities, the greater the risk of generating pollutants and the potential for transporting nutrients and toxicants [60]. The land use of the study area has been severely altered for human activities, particularly for agriculture (Figure 2), exposing its water resources to runoff pollution, which in turn impacts human and environmental health. The dominant land use type, unimproved grassland, occupies 53% of the total catchment area. Unimproved grassland contributes significantly to controlling soil erosion and sediment trapping, but it is not as effective as thickets and bushlands, which are almost non-existent. Thickets and bushlands occupy less than 0.2% of the total catchment area. Cultivated land (rainfed and irrigated), forest plantations and improved grassland (sources of nitrates and phosphates) occupy 39% of the catchment area. Mining and urban areas (sources of toxicants) occupy 6%. Acid mine drainage from coal mining in the province is a major source of environmental damage in Mpumalanga Province [61,62]. The province has the biggest coal deposits in southern Africa. The land use types that have been modified are considered pollution source areas [63]. In most cases, the wetlands have also been modified, mainly for agriculture, reducing their effectiveness in enhancing water quality and providing essential ecosystem services [54,64].

### 2.3 Calculating Polluted Runoff Load

The study developed a simplified model that links runoff generated in the sub-basins with land use areas subjected to anthropogenic changes. The assumption is that these land uses generate pollutants (Equation (1)). The runoff is assumed to collect pollutants from these modified land uses (built-up areas, cultivated lands, mining areas, and modified grasslands). The runoff load transporting the pollutants (soil particulates and dissolved nutrients from agricultural fields) was estimated by calculating the area of each land use type and then multiplying it by runoff generated in each basin coming from the land use type and expressed as:

(1) $$L_{r}=\left(A_{l}\times B_{r}\right)F_{c}$$

where *L*_*r*_ is polluted runoff (m^3^/year), *A*_*l*_ is the area of the land use type that has been altered for human activity and is thus considered a pollutant source land use type (km^2^), *B*_*r*_ is the basin runoff per km^2^ (m^3^/km^2^/year), and *F*_*c*_ is the flow accumulation constraint dataset that determines the exact runoff that discharges into a wetland.

The runoff load, mainly nutrients and toxicants, passes through a wetland as through-flow or is stored for varying periods in wetland storage compartments [65]. Although wetland storage compartments can substantially absorb excess nutrients and toxicants, they have finite boundaries. Once they are full, there will no longer be transfers into these storage facilities. We then developed another equation to calculate the total polluted runoff a wetland can detain (Equation (2)), where the quantity of the runoff load each wetland can detain is calculated as an area percentage of the concentration per volume of the basin and is expressed as:

(2) $$R_{w}=\frac{W_{p}}{100}\times P_{t}$$

where *R*_*w*_ is the total polluted runoff that a wetland can detain (m^3^), *W*_*p*_ is the proportion of a wetland type in relation to the total wetland area of a catchment, and *P*_*t*_ is the total polluted runoff generated in a catchment (m^3^). The *R*_*w*_ also considers the spatial extent of a wetland to process the polluted runoff relative to the amount of polluted runoff and is processed in ArcGIS Pro 3.3.

The two equations (Equations (1) and (2)) can be combined as follows to have a single model to assess wetland capacity to detain polluted runoff. The complete model is presented as follows:

(3) $$Rw=\left(\frac{W_{p}}{100}\times P_{t}\right)\left(\left(A_{l}\times B_{r}\right)F_{c}\right)$$

### 2.4 Runoff Flow Direction and the Upslope Contributing Area

**Figure 3:**
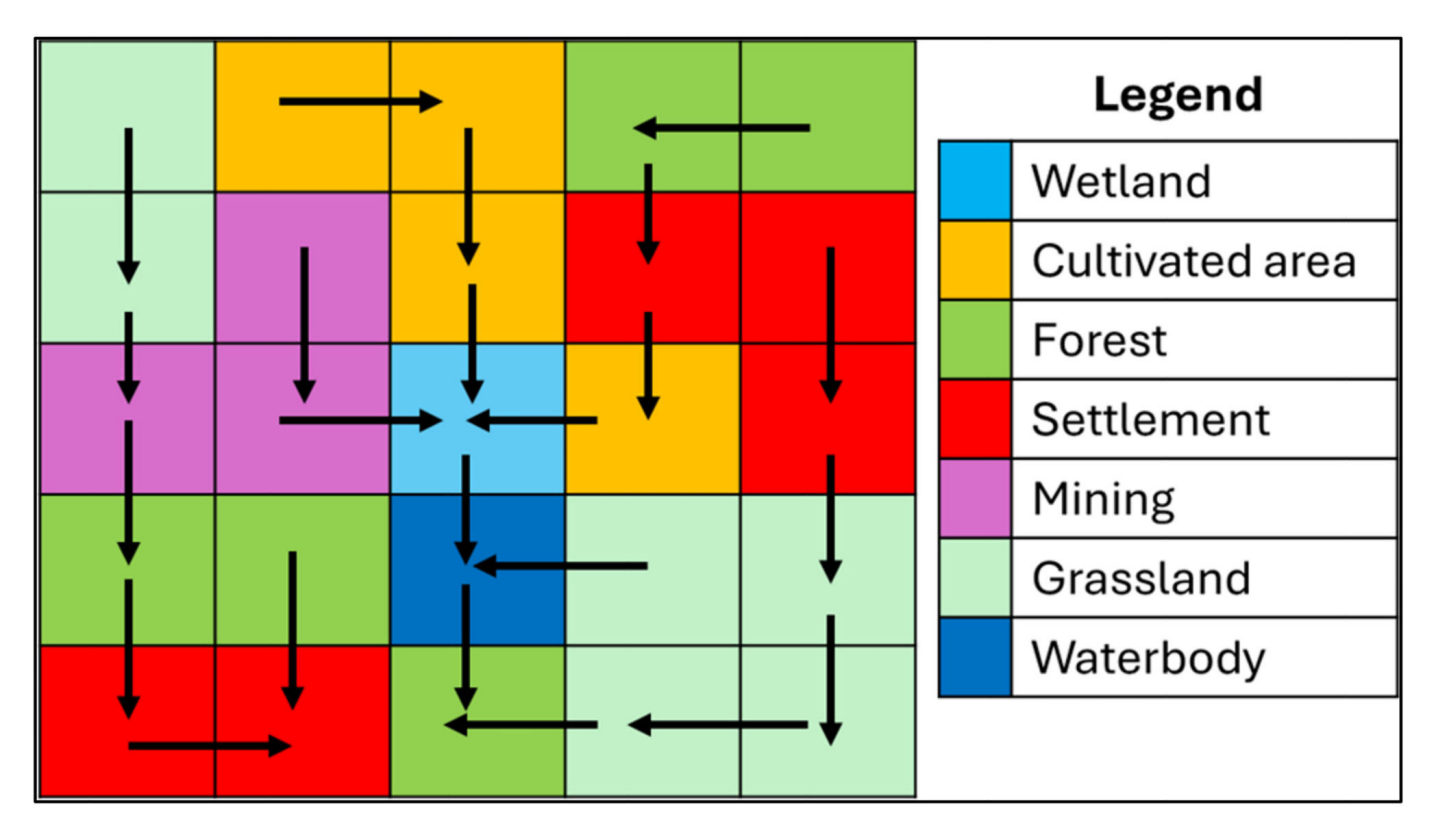
An illustration of how the flow accumulation tool works. The tool was used to constrain and determine the exact runoff that discharges into a wetland.

Runoff flows systematically in a basin, and it follows a defined flow pattern [66]. The Flow Accumulation Tool in ArcGIS Pro 3.3. was used to develop a runoff flow control to determine the quantity of runoff water that flows into each wetland (Figure 3). Thus, the Flow Accumulation dataset was used as a constraint, indicating the runoff discharging into a wetland. This facilitated an understanding of the flow of water into each wetland in sub-basins and the upslope contributing area with polluted runoff. A flow-accumulation dataset (map) indicates areas where runoff will accumulate and, therefore, shows landscapes with the highest inflow of water from an upslope contributing area [67].

The Flow Accumulation Tool generates a spatial model showing densities of runoff flow lines that pass through each pixel (wetland areas), which represents a uniform land unit (which is equal to the upslope contributing area of the slope if multiplied by the map resolution). Figure 3 is an illustration of the results that the Flow Accumulation Tool can produce, where land areas (a) with high flow accumulation are zones of concentrated flow (most likely wetlands) and (b) with a flow accumulation of zero are local topographic highs or ridges which are the sources of runoff.

### 2.5 Ranking of Wetlands According to Filtration Capacity

The wetland types were ranked according to their potential to filter phosphates, nitrates, and toxicants from the runoff load as shown in Table 1. The ranking criteria are derived from the hydrologic benefits rating of wetlands proposed by Kotze et al. [64]. A wetland is ranked 1 (poor) when regarded to have minimal capacity to filter a pollutant type. This means that pollutants will eventually reach waterbodies even if it is temporarily detained for a short time. A rank of 2 (good) means that the wetland is assumed to be able to filter 50% of the pollutants that pass through it, and a rank of 3 (very good) is when a wetland is assumed to have 100% efficiency in filtering pollutants. The authors proposed the filtering percentages by wetland type based on the work of Kotze et al. [64] and from other previous studies that undertook similar work [41,68–70].

### 2.6 Data Sources

The wetland map was obtained from the National Wetland Map 5, which is an improved spatial extent and representation of wetlands of South Africa [54], which can be downloaded at http://opus.sanbi.org/handle/20.500.12143/6917 (accessed on 12 August 2024). The 2020 landcover map was obtained from the Department of Forestry, Fisheries, and Environment (DFFE), available at https://egis.environment.gov.za/sa_national_land_cover_datasets (accessed on 12 August 2024). The mean annual runoff (MAR) dataset was downloaded from the Water Resources dataset (WR90) obtainable at https://www.dws.gov.za/iwqs/wmrq/manual/titles.html (accessed on 12 August 2024). A 30 m resolution Aster Global Digital Elevation Model (Aster GDEM) downloaded from the Earthdata Search at https://search.earthdata.nasa.gov/search (accessed on 12 August 2024) was used to create the flow accumulation dataset that was used to indicate the flow direction and the quantity of runoff that reaches each wetland. All the datasets were processed and analysed in ArcGIS Pro 3.3, a Geographic Information System.

## 3 Results and Discussion

### 3.1 Wetland Typology and Statistics of the Study Area

**Figure 4:**
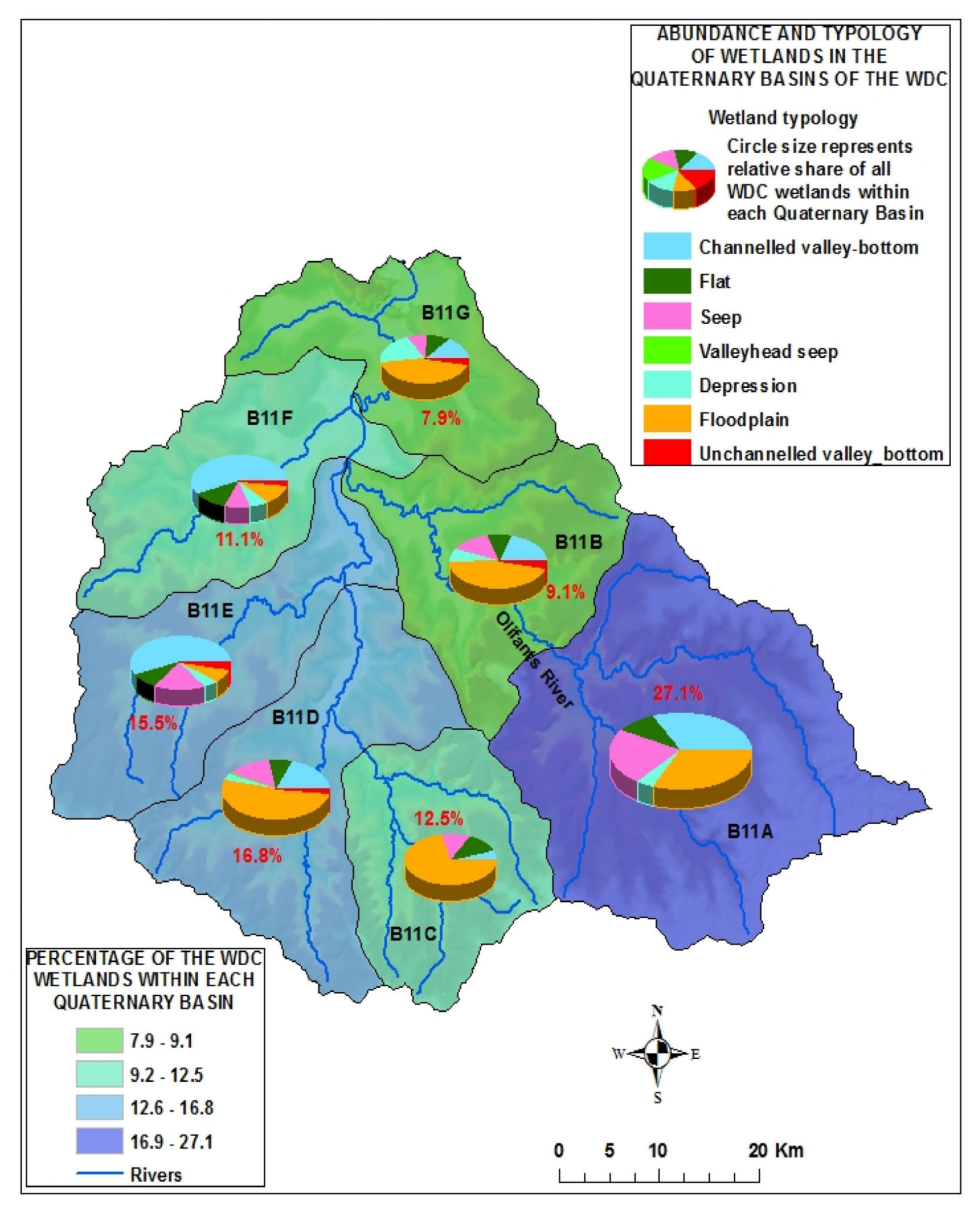
Abundance and typology of wetlands in the Quaternary Basins of the Witbank Dam Catchment.

The distribution of wetland types in each quaternary basin within the catchment is shown in Figure 4. The pie chart size represents the relative share of wetlands in each basin in relation to all the wetlands in the whole catchment, and the chart segments indicate the proportion of the area covered by each wetland type in the respective basin. The percentage value in each basin is the proportion of the wetland area relative to the total wetland area of the whole catchment (also indicated by the colour ramp of each basin). Wetlands occupy 13% of the total surface area of the Witbank Dam Catchment.

### 3.2 Estimating Polluted Runoff Load

The focus was mainly on artificially introduced nitrates and phosphates found in waterbodies originating from agricultural fields and toxicants from mining and urban areas. This study considers these land use types as pollution sources, as they have been modified for human activity. These modified land uses include cultivated land, forest plantations, improved grasslands, mining, and urban areas. Equation (1) was then applied to estimate the annual polluted runoff load in each sub-basin of the catchment (Table 2). The annual runoff from the sub-basin is presented in Table 2.

Polluted runoff originating from each sub-basin is assumed to degrade the waterbodies in that specific sub-basin if the wetland is oversaturated by pollutants beyond its capacity. This implies that water quality enhancement by wetlands occurs within the basin, and once it enters the river network there will be no further sinking of wetlands.

### 3.3 Estimating Polluted Runoff Load Detained by Wetlands

As already alluded to, particulate and dissolved pollutants pass through a wetland as throughflow or are stored for varying periods in wetland storage compartments [65]. Although wetland storage compartments have a substantial ability to absorb excess particulate and dissolved pollutants, they have finite boundaries, and once they are full, there will no longer be transfers into these storage compartments [71,72]. Using Equation (2), the polluted runoff load was estimated as shown in Table 3.

The proportion of a wetland type relative to the total wetland area within the catchment (*W*_*p*_) is shown in the charts in Figure 4. The total polluted runoff generated in a catchment (*P*_*t*_) is given in Table 2. Applying Equation (2), the total polluted runoff (*R*_*w*_) for each wetland type in each basin is calculated and the results are shown in Tables 3 and 4. The whole procedure can also be achieved by using Equation (3). Nitrates and phosphate polluted runoff land use sources are the same (as they result from agricultural activity), unlike toxicants from mining and urban areas. Table 1 shows nitrate and phosphate polluted runoff detained by wetlands, whereas Table 4 shows toxicant runoff detained by wetlands. The sum of polluted runoff for the whole catchment in Tables 3 and 4 gives the same total given in Table 2.

It is assumed that all polluted runoff generated in a catchment is detained by wetlands within that catchment for a certain period, but not all is filtered. Wetland types have different pollutant filtration capacities. Some wetlands are not even capable of filtering certain types of pollutants [64,72]. If a wetland can filter a particular pollutant, it is described as a sink, but if it has no such functions, it becomes a pollutant source [73]. In this study a wetland is regarded as a sink if it has a rank of 2 or 3 (Table 1); that is, it has pollutant-filtering ability. Wetlands ranked 2 or 3 are classified as capable of filtering 50% or 100% of pollutants. Conversely, a wetland is regarded as a pollutant source if it has a rank of 1; it has no pollutant filtration capabilities, thus providing 0% of water quality service. A wetland is considered a sink or filter if the input pollutant is greater than the output, but if the output pollutant is greater than the input, the wetland is considered a pollutant source [64]. Applying the wetland filtration capacity ranking (Table 1) and the data on polluted runoff detained by wetlands (Tables 3 and 4), the quantity of polluted runoff that is not filtered and is eventually discharged into waterbodies is calculated as given in Table 5 using Equations (1) and (2).

### 3.4 Level and Impact of Pollution in the Witbank Dam Catchment

The quantity of polluted runoff that eventually drains into the river system of the Witbank Dam Catchment from quaternary basin B11A was estimated and given in Table 5. The process can be replicated for the other basins. However, the totals for each pollutant type from each basin of the whole catchment are given in Table 6. According to the results shown in Table 2, about 57 million m^3^ of runoff generated in the Witbank Dam Catchment annually are polluted. Of the polluted runoff, 84% originates from cultivated land (irrigated and rainfed) and mines contribute 11%. The rest, 5%, comes from other pollutant sources like urban areas, forest plantations and improved grassland. Runoff from unaltered land use types (unimproved grassland, thicket, and bushland) is excluded, as these land uses enhance water quality by controlling erosion and trapping sediments from reaching the water systems. The totals of polluted runoff from each pollutant type discharged into the river system from each basin are given in Table 5. Table 6 also gives the potential total quantity of polluted runoff water per pollutant type discharged into the river system within the catchment.

The totals for each pollutant type in each sub-basin were used to calculate the total pollutant type and the percentages that are discharged into the whole WDC per annum. A sum of the percentage of the totals of polluted runoff per pollutant type gives a percentage total of 85%. This means a total of 85% of polluted runoff generated in the catchment is discharged into the river system of the catchment annually. The remaining 15% is the only polluted runoff that the wetlands can filter. This may explain why the Witbank Dam (located on the catchment outlet) and the downstream water resources are heavily polluted, risking water use and aquatic life downstream. Several incidents of fish mortality have been recorded in the past years, and most recently the rate of crocodile mortality has been very alarming [36,74].

### 3.5 Validation of the Results

The model results were validated by assessing the contamination levels of As, Cr, Cu, Fe, Mn, Ni, Pb, and Zn taken from samples collected from upstream, midstream and downstream of four tributaries of the Olifants River Basin during a previous study [75] (Table 7). The observed contamination levels of the trace metals were compared with the permissible international levels for waterbodies as per the sediment quality guidelines [76,77]. The overall assessment indicated extremely elevated levels of As, Cr, Mn, and Ni, and some samples were severely enriched and extremely contaminated with As, Cr, and Ni [75]. This is evidence of the predominantly mining and agricultural land uses in the catchment as reported in this study.

Overall, the midstream and downstream sampling points had higher levels of pollutants than upstream sites due to the concentration of pollutants downstream which has endangered aquatic life. This is also evidence of increased anthropogenic activities in the whole basin. The concentrations were even higher in sampling points that were closer to mining, urban and agriculture areas, but steadily decreased with distance from these point sources [75]. Pollutant concentration levels in the Olifants River were also found to be worse than in other rivers in South Africa and other countries [75], except from other known highly polluted water courses that include the Strzyza River in Poland [78], Calore River in Italy [79], Nile River in Egypt [80] and Ipojuca in Brazil [81].

The present study’s results support the findings of previous studies that confirmed the high contamination levels in the Olifants River. The heavily polluted runoff load from the severely altered land use in Olifants Basin has contributed to the Olifants River becoming one of the most degraded rivers in southern Africa, as results from the current study have indicated that over 85% of nutrients and toxicants from coal mines end up in river systems.

### 3.6 Limitations of the Model

The results are only indicative, as other factors like evaporation and export coefficients, among other factors, were not included in this current model. The developed approach only assessed runoff load from anthropogenically modified land covers. It did not consider unmodified natural land cover, as we assumed that unaltered land covers do not generate pollutants. It is also important to note that the effectiveness of wetlands in runoff flow regulation and detention of pollutants depends on their size, placement, and local conditions. Future research can refine the model by considering distinctions between the pollutant loading of different non-natural land cover types. The current model provides the initial phase to develop a more robust approach capable of assessing the capability of wetland types to filter pollutants and guide strategic policy decisions to reduce pollutant loads from anthropogenically modified land covers. One such policy decision would be to indicate spatial areas in the catchment where wetlands would benefit more in enhancing water quality due to high pollution levels. Also, we assumed that all the wetlands in the catchment are in good condition to enhance water quality, yet this may not be what is on the ground, as some wetlands could have been severely degraded by mining and other anthropogenic activities and no longer offer the ecosystem services at the same level as when they were healthy. A comprehensive fieldwork may be required to assess the clear state of the wetlands. The wetland state is another factor that needs to be included in the refined model. Areas for future research include the role of denitrification in pollutant removal and the uptake of pollutants by plants as nutrients. This current study only focused on the capability of wetlands to detain runoff pollutants.

## 4 Conclusions

This study developed a simplified approach to assess the impact of polluted runoff load on wetland health to continue offering optimum ecosystem services. The approach facilitated the assessment of wetland value in enhancing water quality and ensuring human and environmental health. Surface runoff has been considered a standard to quantify nonpoint source pollution, as it is responsible for transporting nutrients and toxicants into river systems. The method enabled estimating the polluted runoff discharged into the river system, giving results that are consistent with the water quality challenges in the Witbank Dam Catchment and the downstream Limpopo River basin which has seen a rise in crocodile and fish mortality. The heavily polluted runoff load from the severely altered land use in the catchment has contributed to the Olifants River becoming one of the most degraded rivers in southern Africa. Over 85% of nutrients and toxicants from coal mines end up in river systems, risking human and environmental health. The applied method allowed identifying pollutant source areas, making it a valuable tool for decision-makers, especially for remedial intervention. Although wetlands in the Witbank Dam Catchment are playing a key role in enhancing water quality, huge quantities of polluted runoff are still discharged into the river system of the catchment, as the wetlands are unable to cope with the heavily polluted runoff load. Although wetlands have a substantial ability to absorb excess pollutants, they have finite boundaries, and once they are full, they will no longer be able to absorb any further pollutants. The excess is discharged into the river system.

## Figures and Tables

**Table 1:** Nutrient and toxicant regulatory services provided by wetlands.

Wetland Type	Phosphates	Nitrates	Toxicants
Channelled valley bottom	2	2	2
Flat	1	2	2
Seep	1	3	2
Valley-head seep	1	3	3
Depression	1	2	2
Floodplain	3	2	2
Unchannelled valley bottom	2	2	2

Note(s): 1—Poor, 2—Good, 3—Very good.

**Table 2:** Contaminated runoff produced in each quaternary basin per land use type in the Witbank Dam Catchment.

Quaternary Basin	Area (km^2^)	Runoff (10^3^ m^3^/km^2^/Year)	Polluted Runoff Volume from Each Land Use/Cover (103 m^3^/Year)					Total Polluted Runoff (10^3^ m^3^/Year)
			Cultivated Land	Forest	Improved Grassland	Mining	Urban	
B11A	946.05	38.89	15,410.62	177.76	117.92	0.00	23.86	15,730.2
B11B	435.43	36.16	7218.41	71.93	8.96	927.66	195.13	8422.1
B11C	387.07	33.11	5340.34	9.77	0.00	0.00	0.04	5350.2
B11D	551.20	30.08	5823.63	35.78	43.14	872.24	320.37	7095.2
B11E	466.03	32.24	6221.80	102.98	69.70	1125.53	65.88	7585.9
B11F	430.34	34.26	5340.15	248.98	134.02	1486.07	94.10	7303.3
B11G	367.77	35.84	2655.54	350.31	194.11	1763.38	615.17	5578.5
Total	3583.9		48,010.5	997.5	567.8	6174.9	1314.6	57,065.3

**Table 3:** Total nitrate- and phosphate-polluted runoff detained by wetland types in each basin.

Wetland Type		N- and P-Polluted Runoff Detained by Wetland Types (10^3^ m^3^/Year)						Total (WDC)
	B11A	B11B	B11C	B11D	B11E	B11F	B11G	
Channelled valley bottom	5267.01	1395.06	251.05	1087.36	3527.45	3194.71	418.13	15,140.77
Depression	677.05	391.52	50.74	157.20	333.77	450.05	550.75	2611.08
Flat	1515.88	805.06	606.41	563.47	554.15	686.10	360.16	5091.25
Floodplain wetland	5221.65	3427.14	3727.96	3052.29	401.37	599.57	1475.57	17,905.55
Seep	2915.11	1054.23	667.15	888.99	1399.28	660.56	318.92	7904.24
Unchanneled valley bottom	49.13	213.01	12.65	90.84	166.13	108.25	74.73	714.74
Valleyhead seep	60.42	12.82	34.03	62.15	12.48	23.85	1.96	207.72
Total	15,706.26	7298.84	5350.00	5902.31	6394.63	5723.09	3200.22	49,575.35

**Table 4:** Total toxicant-polluted runoff detained by wetland types in each basin.

Wetland Type	Toxicant-Polluted Runoff Detained by Wetland Types (10^3^ m^3^/Year)							Total (WDC)
	B11A	B11B	B11C	B11D	B11E	B11F	B11G	
Channelled valley bottom	8.00	214.59	0.00	219.70	657.23	882.06	310.80	2292.39
Depression	1.03	60.22	0.00	31.76	62.19	124.26	409.37	688.84
Flat	2.30	123.84	0.00	113.85	103.25	189.43	267.71	800.39
Floodplain wetland	7.93	527.17	0.03	616.72	74.78	165.54	1096.80	2488.97
Seep	4.43	162.16	0.00	179.62	260.71	182.38	237.05	1026.36
Unchanneled valley bottom	0.07	32.77	0.00	18.35	30.95	29.89	55.55	167.58
Valleyhead seep	0.09	1.97	0.00	12.56	2.33	6.59	1.46	24.99
Total	23.86	1122.72	0.04	1192.56	1191.44	1580.15	2378.74	7489.52

**Table 5:** Nutrient and toxicant runoff that eventually enters the river system in B11A (10^3^ m^3^/year).

		Phosphates		Nitrates		Toxicants		
Wetland Type	Total P and N Runoff	Polluted Outflow	Rank	Rank	Polluted Outflow	Total Tox Runoff	Rank	Polluted Outflow
Channelled valley bottom	5275.01	2	2637.51	2	2637.51	8.00	2	4
Depression	678.08	1	678.08	2	339.04	1.03	2	0.51
Flat	1518.19	1	1518.19	2	759.10	2.30	2	1.15
Floodplain wetland	5229.59	3	0.00	2	2614.80	7.93	2	3.97
Seep	2919.54	1	2919.54	3	0.00	4.43	2	2.22
Unchanneled valley bottom	49.21	2	24.61	2	24.61	0.07	2	0.04
Valleyhead seep	60.51	1	60.51	3	0.00	0.09	3	0
Total polluted outflow	15,730.12		7838.44		6375.06	23.86		11.89

**Table 6:** Polluted runoff discharged into the river network of the Witbank Dam Catchment.

Quaternary Basin	Type of Pollutant Runoff (10^3^ m^3^/Year)		
	Phosphate Runoff	Nitrate Runoff	Toxicant Runoff
B11A	7838.44	6375.06	11.89
B11B	3068.67	3115.9	560.39
B11C	1490.19	2324.47	0.02
B11D	2260.91	2455.59	584.81
B11E	4146.48	2491.46	594.62
B11F	3472.1	2518.91	786.79
B11G	1478.23	1439.69	1288.66
Total (WDC)	23,755.02	20,721.08	3827.18
Percentage (%) of total polluted runoff	42%	36%	7%

**Table 7:** Average pollutant concentrations in the Olifants River and the average shale values and sediment quality guidelines (SQG) values.

Sampling Point	AS	Cr	Cu	Fe	Mn	Ni	Pb	Zn
BL1	50.8	41.5	63.6	25,333.0	685.3	109.9	7.5	42.8
BL2	51.0	80.4	74.0	28,108.8	949.8	115.1	7.2	48.3
BL3	45.0	108.0	63.4	46,210.0	1299.0	281.7	7.4	38.6
MH1	6.6	260.5	21.7	12,001.5	567.8	64.7	5.9	16.2
MH2	8.0	245.0	16.4	74,664.5	2581.5	49.2	8.5	20.3
MH3	4.8	416.3	20.3	34,761.0	682.3	72.0	10.0	54.5
SL1	3.8	61.8	0.0	14,400.0	254.5	79.5	0.0	11.0
SL2	2.0	52.5	0.0	15,000.0	270.5	144.7	0.0	29.5
SL3	1.7	46.8	0.0	12,700.0	209.0	88.3	0.0	29.0
ST1	0.9	106.3	25.3	177,173.8	1167.8	16.6	5.4	89.1
ST2	0.4	2252.8	13.7	133,291.8	1560.8	92.0	4.1	47.5
ST3	1.9	1096.3	21.2	135,981.5	1783.8	119.5	17.0	92.0
Av. Shale value *	13.0	90.0	45.0	47,200.0	850.0	68.0	20.0	95.0
SQG*	5.9	37.3	35.7					

* Note(s): Turekian and Wedepohl [77]; SQG, Sediment Quality Guideline [76]. Source: Addo-Bediako [75].

## Data Availability

All data that support the findings of this study are included within the article.
